# An Essay upon the Treatment of the Deep and Excavated Ulcer, with Cases

**Published:** 1830-01-01

**Authors:** 


**AN ESSAY UPON THE TREATMENT OF THE DEEP AND EXCAVATED ULCER: WITH CASES. By *Richard Anthony Stafford*, Member of the Royal College of Surgeons, &c. Octavo, pp. 72. London, 1829. Price Five Shillings.**

THE comparative absence of "original communications" from the journals devoted or adapted to their reception, has been very apparent of late years. Two causes have conspired in producing this deficiency of such articles in the market:—first, the degree of scepticism which just or unjust is certainly extended by the public to cases detailed by the parties interested; and secondly, the obloquy, ribald jest, and foul language with which many respectable contributors have been assailed by the low-bred scurrilous fellows that adorn a certain portion of the medical periodical press. These circumstances have operated in strangling much trash that would otherwise have seen the light, and so far they have done good; but they have also kept back very useful information, and deterred many diffident well-informed men from venturing into the gladiatorial arena of the journals. They have also operated in another way and induced men to publish in octavos and duodecimos what might have done well enough for the substance of a letter to an editor, but cuts a sorry figure when diluted into a book of some seventy or eighty pages. There is scarcely a more unpleasant task than that of reviewing such a publication, for justice requires the veil to be withdrawn which private feeling would hang before the weaknesses of the author. It is a duty however which the conductor of a critical journal owes to that public which honours him by its support, and coute qui coute it must be performed.

We were led to make these preliminary remarks from perusing the little volume of Mr. Stafford. We think he has acted unwisely in making a five-shilling book out of the materials at his disposal, and *wire-drawing* the substance of some four or five pages into seventy-two. The ductility of his matter may be great, but like certain metals it is weakened in proportion as it is lengthened. The object of Mr. Stafford is to recommend the employment of melted wax in the treatment of deep and excavated ulcers of various kinds, and one or two brief extracts will shew the practice quite as well as the perusal of the whole publication. The composition of the wax is as follows:—four parts of white wax, and one of Venice turpentine; it can be bought at Field's and Son, Wigmore Street.

"The treatment which I am about to recommend is extremely simple; and we shall find, upon inquiry, that its action is founded on just principles, and according to the laws which nature herself pursues. It consists in pouring into the excavation melted wax, of an extremely adhesive quality, and just at that temperature when it is on the point of cooling, and will immediately become solid in the wound. In this manner the under surface of the wax, when cold, comes into close contact with the general surface of the ulcer, and the whole excavation is filled by it. Before employing it, however, it is necessary that one or two precautions should be taken: first, in order to clean the sore, as much of the pus as possible which rests upon it should be absorbed by dry lint; and secondly, in order to avoid burning the patient, the wax should be at that point of heat which is called by chandlers *setting;* that is, a portion of it should cling to the sides of the vessel in which it was melted, and the rest should begin to thicken, and have somewhat of an opaque appearance. In this state it will not be at much more than blood heat, and it can be used with perfect safety. It is advisable, however, even when so far cooled, that a brush be dipped in it, and that the wax be allowed to drop from that into the sore. After the wax becomes perfectly solid in the ulcer, a strip or two of adhesive plaister may be applied over it, to keep it in its situation; when it may be left until it requires to be dressed again, which will be on the third day after its application. By pursuing this method of treatment, it will be found that healthy granulations will be produced, and appear upon the whole surface of the sore; that it will contract; and that the healing process will proceed very rapidly." 6.

"With regard to the progress by which a sore heals, according to this or any other method of treatment, it is impossible to lay down any precise rule or time, as this must in some measure depend upon its character, its size, the health of the patient, &c.; but the stages by which the reparative process is usually carried on, when treated with the wax, is as follows:—On the removal of the first dressing, the sore generally presents a cleaner surface, being more reddened; and sometimes even in the early stages granulations are distinguishable. After the second dressing they are commonly spread over the whole surface of the sore; on the third they partly fill up the cavity, which is much contracted; on the fourth it appears still less; and so on until it is completely closed, and then the skinning process commences. During the course of healing, likewise, it may be observed that the granulations are smaller, more compact, and more florid. The cicatrix also presents a more even surface; it is of a firmer texture, less tender, and does not appear so likely to break out again as the scars of those ulcers which have not been treated according to this plan.

"The superiority of this plan of treatment is, that the sore is healed much more quickly, being, in fact, so rapid, that it is accomplished in one-third of the time usually occupied, and with much greater certainty than where the common methods are employed. It succeeds also where no other remedy will, as may be seen by the cases. It excludes the air from the wound, shielding it at the same time from external objects; it makes equal pressure upon its surface, and thus supports its tender vessels; and it imitates the process which nature herself pursues, the healing of a sore by scabbing. All these are of great use; for, in the first place, by the exclusion of the air, much irritation and pain is avoided; in the second, by the support it gives to the tender vessels of the ulcer, the cicatrix is of a more firm and solid texture; and in the third, by the scabbing process, it is healed in a more regular manner. In addition to these advantages, the pain, when the wax is upon the sore, is so little, that many of the patients have informed me that they have been almost unconscious, not only of its presence, but even of the existence of the sore itself.

"The cases where the plan of treatment I have recommended might be advantageous are, the open and excavated Bubo; ulcers of the legs; indolent scrofulous sores; excavations in the flesh, in consequence of sloughing phagedena; ulcers situated over large arteries; sinuses, and fistulous passages, that have been laid open; the sores left by extensive burns, broken chilblains, and, in short, those of any depth, from whatever cause they may arise. In most of the cases I have just enumerated, the wax has been employed; but more particularly in ulcerated legs, open Buboes, and scrofulous sores. In these, of whatever character or description they may have been, the treatment has succeeded, and the healing process has been forwarded with greater rapidity than where the ordinary applications have been used. Its utility does not appear to be confined to any one particular state of the ulcer. When it is extremely foul, and even covered by a sloughy matter, as in Butler's case, on the removal of the wax it has presented a clean surface. Where the ulceration has been extending, its progress has been immediately arrested, and it has shewn a disposition to heal. When the sore has been connected with varicose veins on the leg, it has been attended with equal advantage; and when its character has been of so languid and indolent a nature, that no remedy could excite a healthy action, the stimulus and support of the foreign body, that is, the wax, has, at it were, like a charm, produced strong and florid granulations.

"Although there are very few species of ulcers where this method of treatment might not be successful, yet it offers peculiar benefit in many cases which before defied all other remedies: for instance, in sores situated over large arteries, where there is a danger of the ulcerative process being continued into the vessel. Such cases are not uncommon; and, to my own knowledge, several patients have died in consequence of every application having proved ineffectual to stop its progress. In these instances it is customary to apply stimulating remedies; but every practitioner must have observed, that such dressings have too often accelerated the catastrophe they have intended to ward off. Here, then, the use of the wax might be of singular advantage, by producing healthy granulations, and, at the same time, protecting the artery. The same plan of treatment might likewise be resorted to in all extensive sores, such as burns; and thus not only might they be made to heal more quickly, but they would likewise be shielded from objects around them. There are some species of ulcers, also, whose peculiar character it is to spread; for instance, herpetic sores, noli me tangere, and cancerous ulceration; and if the principle which I have pointed out as to the action, which this plan of treatment induces in the ulcer, be correct, it might possibly be of infinite service in these cases, in putting an end to, or at least stopping the progress of, the ravages of the disease; and more particularly when it is extending itself into parts so full of blood vessels, that there is reason to apprehend the death of the patient from hæmorrhage. I have not had an opportunity of employing it in any of these cases, excepting in cancerous ulceration, where its effects in producing granulations (which will be seen by the cases) was extraordinary; consequently, I am unable to bring forward any facts to establish its utility, and therefore I merely offer these remarks as a suggestion.

"In conclusion, it is almost needless to say, that whatever local treatment may be applied to an ulcer, it is necessary to attend to the health of the patient; and although in most of the cases where the wax was used, only purgatives were occasionally given, yet, if the health be deranged, constitutional treatment must likewise be resorted to." 21.

A great number of cases are detailed which are more adapted for making a book than enriching a journal. We refer the curious to the work itself,—

Where twice ten thousand shake the labouring field.

